# *Alliaceae*-Derived Supplementation Improves the Severity of COVID-19 Symptoms among Elderly Nursing Home Residents

**DOI:** 10.3390/foods13172718

**Published:** 2024-08-27

**Authors:** Alberto Vázquez-Blanquiño, Lucía Pérez-Rodríguez, Ana Alberola-Romano, María Martínez-Pérez, Alberto Baños, Germán O. Gómez-Fernández, Carlos Gracián, Juristo Fonollá, Federico García

**Affiliations:** 1 Clinical Microbiology Department, Hospital Universitario Clínico San Cecilio, 18016 Granada, Spainluciapr1996@gmail.com (L.P.-R.); anaalberolaromano@gmail.com (A.A.-R.);; 2 Pharmacy Department, Hospital Universitario Clínico San Cecilio, 18016 Granada, Spain; mariamape30@gmail.com; 3 DMC Research Center, Camino de Jayena, 82, 18620 Granada, Spain; abarjona@dmcrc.com; 4 Department of Sciences, Public University of Navarra, 31006 Pamplona, Spain; german.gomez@unavarra.es; 5 Nursing Home “Residencia de Mayores Claret”, 18011 Granada, Spain; cgracianalcaide6@gmail.com; 6 Department of Nutrition and Food Technology, University of Granada, 18071 Granada, Spain; 7 Instituto de Investigación Biosanitaria (ibs.GRANADA), 18014 Granada, Spain; 8 Centro de Investigación Biomédica en Red en Enfermedades Infecciosas (CIBERINFEC), ISCIII, 28029 Madrid, Spain

**Keywords:** *Alliaceae* supplementation, nursing home intervention, COVID-19, SARS-CoV-2, garlic, onion

## Abstract

This study investigates the effect of daily consumption of a concentrated garlic and onion extract on COVID-19 symptoms among elderly nursing home residents. Volunteers consumed a daily capsule of the concentrated powder rich in organosulfur compounds over 36 weeks during lunch. The incidence and severity of COVID-19 symptoms between the treatment and control groups were compared, along with monitoring the safety of consumption, incidence of other diseases, and medicine usage. The treatment group showed a significant reduction in both the number and severity of COVID-19 symptoms compared to the control group, with no significant adverse effects observed. No significant reduction in symptom duration was detected. This study provides preliminary evidence that concentrated garlic and onion extract may aid in the treatment of COVID-19 among older adults. These findings suggest potential public health benefits, emphasizing the need for further research to explore the immunomodulatory properties of these natural compounds.

## 1. Introduction

According to the World Health Organization (WHO), the SARS-CoV-2 pandemic has caused over 6 million deaths worldwide, with more than 771 million reported infections. Additionally, more than 13 million vaccines have been administered, demonstrating a significant reduction in both the number of hospitalizations and severe cases [1].

During this pandemic, numerous treatments have been used to combat the disease: antivirals, anti-inflammatory drugs, monoclonal antibodies, etc. [2]. Currently in Spain, according to the Spanish Agency for Medicines and Health Products (AEMPS), antivirals such as remdesivir, nirmatrevir/ritonavir, monupiravir, and monoclonal antibodies such as sotrovimab, casirivimab/imdevimab and cilgavimab/tixagevimab are approved. These drugs are typically authorized for use in severe or critical patients, high-risk patients, and those with mild/moderate disease [3]. However, certain plant-derived products have also been extensively studied as complementary and/or preventive treatments for COVID-19 [4,5]. Some of their bioactive components possess various phytotherapeutic properties, such as antiviral and anti-inflammatory properties, making them suitable for treating infections caused by SARS-CoV-2 [6]. In this regard, the literature has reported, among others, the potential therapeutic effects of curcumin [7], cinnamon [8], ginger [9], and garlic [10]. Specifically, this study will focus on the combination of garlic (*Allium sativum* L.) and onion (*Allium cepa* L.) extracts. Both are characterized by containing a large number of phytochemicals, especially sulfur-rich compounds. Among these compounds, the main ones include thiosulfinates and thiosulfonates such as diallylthiosulfinat (allicin), propyl-propane-thiosulfinate (PTS), and propyl-propane-thiosulfonate (PTSO); and sulfides such as diallyl disulfide (DADS) and diallyl trisulfide (DATS), among others, to which several properties are attributed, such as anti-inflammatory and antibacterial effects, or as modulators of the intestinal microbiome [11,12]. These substances are mainly responsible for the antimicrobial activity they exhibit, highlighting in this case the antiviral activity [13,14] that has already been evaluated against various human pathogenic viruses such as influenza B, HIV (type 1), vesicular stomatitis virus, herpes simplex virus (types 1 and 2), Coxsackie virus, and gammaretrovirus [15].

The disease caused by SARS-CoV-2 tends to develop more severely in certain population groups such as the elderly, individuals with type 2 diabetes mellitus, obesity, hypertension, smokers, males, etc. Generally, the older population is more vulnerable to infectious diseases due to a decline in their overall health and particularly in their immune system. The same holds true for COVID-19, with age being the most significant risk factor for developing severe illness and associated death [16].

In this study, our aim was to evaluate the effect of daily consumption of a combination of powdered garlic (*Allium sativum* L.) and a concentrated onion extract (*Allium cepa* L.), and to study their impact on COVID-19-related symptoms in a population of institutionalized elderly people diagnosed with this disease.

## 2. Materials and Methods

### 2.1. Ethics, Approval, and Consent

This research was conducted in accordance with the Ethical Principles for Medical Research involving human subjects outlined in the Declaration of Helsinki and its subsequent amendments. It also adhered to the Good Clinical Practice guidelines specified by CPMP/ICH/135/95, ISO 14155 [17], and all relevant Spanish regulations. The study protocol received approval from the Regional Ethical Committee in Granada, Spain, and was registered with the US Library of Medicine (http://www.clinicaltrials.gov, accessed on 1 February 2022) under the ID NCT04647071.

Before starting the study, the participants were selected by the medical team of the residency according to their characteristics and were invited to participate. If they accepted the invitation, the family members of all participants were also informed and asked to give their consent as well. Written informed consent was obtained from all subjects before including them in the study.

### 2.2. Subjects and Study Design

This is a retrospective study, continuation of a previous study that evaluated the effect of consuming a concentrated garlic and onion extract on respiratory diseases of infectious origin [18]. After a three-month washout period (summer months), the volunteers in the control group began taking a capsule of the product at lunch for 36 weeks, and the volunteers who had taken the product did not take it during this time.

**Figure 1:**
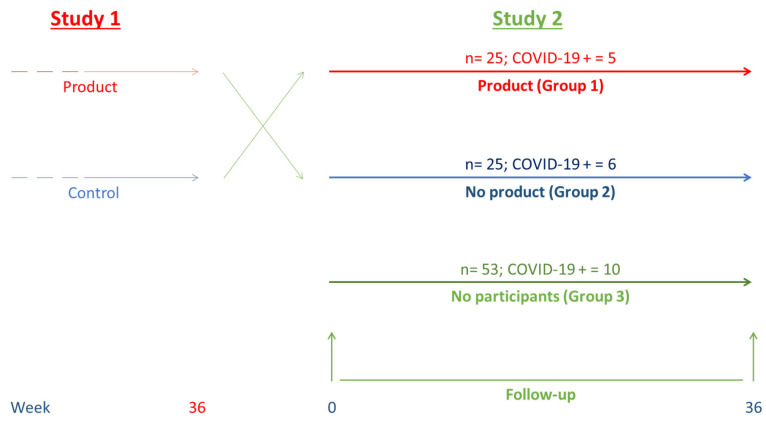
Study design.

The first study involved 65 healthy elderly volunteers of both sexes, all of whom lived in the elderly care home Residencia de Mayores Claret in Granada (Spain). The participants were over 65 years of age and had been vaccinated against influenza, meeting the established inclusion criteria [18]. The study was completed by 50 volunteers and for ethical reasons, it was decided to give the study product to the volunteers in the control group during the same time. Thus, the 25 volunteers in the control group who completed the study began taking one capsule a day of the study product during lunch (group 1) and the remaining 25 volunteers stopped taking the product and did not take anything. All 50 volunteers were followed during this time. However, only 11 of these volunteers were infected with SARS-CoV-19, five in the intervention group and six in the control group, along with 10 others who did not participate in the study. These 21 volunteers were followed for analysis of symptoms caused by the infection. In addition, the 10 residents who were not participating in the study and had a confirmed episode of COVID-19 were asked for permission (informed consent) to collect information about related symptoms (Figure 1). All participants had been vaccinated against both the flu and pneumonia, and they received two doses of the Pfizer vaccine for SARS-CoV-2. The fact that they were not vaccinated was considered an exclusion criterion for the study.

All study participants followed the same dietary regime throughout the intervention.

### 2.3. Intervention

The active product (Aliocare^®^, DOMCA SAU, Granada, Spain) contained concentrated onion extract (86 mg) standardized in organosulfur compounds derived from propiin (10 mg per capsule), garlic powder (14 mg), and microcrystalline cellulose (9892-Capsucel^®^, Laboratorios Guinama, La Pobla de Vallbona, Valencia, Spain), with a total weight of 450 mg. The product is administered in hydroxypropyl methylcellulose capsules (Solchem^®^, Solchem, Barcelona, Spain). The medical team at the nursing home was responsible for administering one capsule daily to each volunteer during lunch over the course of 36 weeks, ensuring proper ingestion.

Dehydrated garlic powder was utilized in this study, and it was thoroughly analyzed to confirm the absence of vitamins and thiosulfinate compounds, including allicin. The product exhibited a variable composition of sulfur-containing compounds, primarily diallyl disulfide and diallyl trisulfide, which together accounted for approximately 1.5% of the total content.

### 2.4. Clinical Parameters

The symptoms followed for COVID-19 were the same as those studied in the first one: fever (temperature), cough (type), shortness of breath, stuffy nose, chest pain, sore throat, headache, bone/muscle pain, fatigue/exhaustion, nausea/vomiting, diarrhea, lack of appetite, loss of smell/taste, and sleeping problems.

The variables studied were number of symptoms, duration of symptoms, and antigen-positive days. The medication taken and the time of treatment was also analyzed.

All study subjects were followed up by measuring their weight and blood pressure. In addition, volunteers who were taking or had taken the product (groups 1 and 2, respectively) were followed up to check the safety of the product and to study possible adverse effects from its consumption.

### 2.5. Statistical Analysis

For the initial values of parameters used as controls such as weight, body mass index (BMI), and habits, T-tests and Kruskal–Wallis tests were carried out with a significance level of 0.05, as a method of testing for null differences between the control, treated, and treatment groups, prior to the experiment.

The analysis was carried out based on one-way parametric analysis of variance (ANOVA) after testing for normality assumptions (*p*-value < 0.01) and homogeneity of variances using Shapiro–Wilks and Levene tests, respectively. Additionally, multiple comparisons were carried out using Fisher’s LSD tests. For the number of symptoms, the Kruskal–Wallis method and subsequent Z-test were applied with a significance level of 0.05.

## 3. Results

The first study was performed during the height of the COVID-19 pandemic in Spain, from November 2020 to July 2021, characterized by a considerable increase in the number of infections and a markedly high mortality rate. Despite the extreme preventive measures implemented in our study residence, some cases of respiratory diseases of infectious origin, including SARS-CoV-2 infection, were reported during this period. However, confirmation of these cases was based solely on clinical diagnosis due to the saturation of the health system at that time, which prevented the performance of analytical tests such as antigen testing, PCR, or antibody measurement.

As shown in Table 1, in the first study, a total of 4 COVID-19 events were diagnosed in the treatment group (with one volunteer being diagnosed twice) and 13 in the control group (with two volunteers being diagnosed twice). Volunteers in both groups resided in the same areas and were thus equally likely to be infected. The lower number of COVID-19 cases in the treated group (3800 times less than the control group; *p* = 0.032) may be attributed to the absence of analytical test confirmation, as mentioned earlier. In the previous study [19], volunteers who received the product reported fewer symptoms related to respiratory diseases, suggesting that the clinical diagnosis of COVID-19 was lower in this group.

However, the second study was conducted during the seventh wave of the pandemic, which coincided with the circulation of the Omicron variant of SARS-CoV-2. On this occasion, there were new cases of COVID-19 in the nursing home that could be confirmed by analytical methods. In addition, when all these infections occurred, the medical service decided to test all residents (not only those who participated in the study) for the antigen. All positive cases were confirmed by PCR testing. Family members and residents who tested positive and were not study volunteers were asked for informed consent so that their symptomatic data could be collected.

In total, 21 cases of COVID-19 were confirmed and were considered active until they tested negative on antigen testing, and none of these cases died from COVID-19. Table 2 classifies the patients who tested positive for COVID-19 into 3 groups: those who were being treated during the study (*n* = 5), those who had been treated during study 1 (*n* = 6) and those who had never been treated with the product (*n* = 10). The characteristics of the population that was followed are described in this table. It can be seen that the population studied is homogeneous in terms of characteristics and habits. No significant differences were observed between the different study groups.

Table 3 shows the statistical study of the variables analyzed. Of the 5 volunteers in the treatment group who tested positive for COVID-19, only 2 reported having a slight cough. The other 3 did not report any symptoms. However, the symptoms experienced by the volunteers in the other 2 groups, who were not taking the product during this study, were significantly higher; being the most common cough (13/16), fatigue/exhaustion (8/16), lack of appetite (6/16), sore throat (6/16), and bone/muscle pain (6/16).

The duration of symptoms was similar in all 3 groups. However, the time in which the volunteers tested positive for antigens was significantly shorter in the group that stopped taking the product several months earlier. Moreover, the use of medication to treat these symptoms and the time of use of medication was the same for the 2 groups not taking treatment. The treatment group did not use any medication to treat symptoms.

Table 3 also indicates that the proportion of those infected by the virus in the three groups was similar (20%, 24%, and 19%, respectively). If the diagnosis had been made clinically, the result would probably have been zero for the treatment group. We believe this was the case in the first study.

In terms of the safety profile, no adverse effects were detected in patients taking the product. In addition, no significant changes in weight and blood pressure measurements were observed.

These results are consistent with those obtained in study 1, both for those diagnosed with COVID-19 and for other respiratory diseases of infectious origin [18].

## 4. Discussion

This retrospective study, building on our previous research, provides significant insights into the effects of consuming concentrated garlic and onion extract on the incidence and severity of COVID-19 among elderly residents in a nursing home setting. The continuation of the study during the seventh wave of the pandemic, marked by the emergence of the Omicron variant, offered a unique opportunity to further assess the potential protective effects of these *Allium*-derived compounds against a more transmissible variant of the virus. By potentially reducing virus transmissibility and aiding in faster recovery, these natural compounds may offer significant public health benefits.

Our results indicate a notable reduction in the incidence of COVID-19 among participants who consumed garlic and onion extract, consistent with the results observed in our initial study [18]. This result strengthens the hypothesis that specific organosulfur compounds present in garlic and onion may confer an enhanced immune response against viral pathogens, including SARS-CoV-2. The significantly shorter duration of antigenic positivity observed in the product pre-treated group highlights a possible protective effect against infection, in this case with SARS-CoV-2.

Furthermore, interestingly, the duration of COVID-19 symptoms was similar in all groups in our study, but the group that actively consumed the extract during the study period reported fewer and milder symptoms related to COVID-19 infection. This observation is consistent with the existing literature on the possible antimicrobial and, in particular, antiviral activities of *Alliaceae* compounds, which have been proposed to exert their effects through different mechanisms [19,20]. These observed effects highlight the potential human health benefits of consuming garlic and onion extracts.

Our study did not conclusively demonstrate direct antiviral activity of these compounds against SARS-CoV-2. However, the reduction in the number of symptoms and in the period of virus shedding could be explained through the anti-inflammatory and immunomodulatory properties of organosulfur compounds from *Allium* species. The compounds responsible for the immunomodulatory, anti-inflammatory and antiviral activity of garlic include thiosulfinates (allicin), S-allylcysteine sulfoxides (alliin) and ajoenes (E- and Z-ajoene), among others [13]. In the case of onion, the most prevalent compound responsible for its activity would be propyne (S-propyl-L-cysteine sulfoxide), which, upon reaction with the enzyme alliinase, is transformed into derivatives of this molecule, such as propylpropane thiosulfinate (PTS) and propylpropane thiosulphonate (PTSO) [21]. These compounds present in these plants have been tested in numerous studies against SARS-CoV-2 infection, both in silico [22,23,24], in vitro [25] and in vivo [26,27,28,29]. In addition, some of the studies show quite interesting results. This is the case of Wang et al., a multicenter study in 3 hospitals where 97 patients received garlic essential oil combined with conventional treatment. They found that, compared to the control group, patients treated with garlic showed shorter duration of symptoms, shorter time to negative results in nucleic acid screening tests and shorter time to improved imaging tests [27]. These are similar results to those obtained in the present study, although with a different population.

The inflammation caused by SARS-CoV-2 infection is a critical factor in the progression and severity of COVID-19 [30]. This viral infection triggers an exaggerated immune response, often referred to as a cytokine storm, characterized by the excessive release of pro-inflammatory cytokines such as IL-6, IL-1β, and TNF-α [31,32]. This hyper-inflammatory state can lead to acute respiratory distress syndrome (ARDS), multi-organ failure, and, in severe cases, death. Understanding the mechanisms behind this inflammatory response is crucial for developing targeted therapies to mitigate its impact and improve patient outcomes [33]. In this situation, the immunomodulatory and anti-inflammatory effect of certain compounds present in garlic and onions could be useful in preventing these immunological phenomena. It appears that this mechanism is due, among other things, to the fact that it significantly improves the levels of T-helper lymphocytes, cytotoxic T-lymphocytes and NK cells. In addition, it has also been shown to play an important role in the negative regulation of leptin (a pleiotropic hormone with important effects on the immune system), the leptin receptor, TNF-a, IL-6, and proliferator-activated receptor gamma (PPAR-g) [10]. Additionally, in previous studies, we have demonstrated that these organosulfur compounds derived from propiin in onion, such as PTS and PTSO, exhibit immunomodulatory properties in cell lines, with significant reductions in pro-inflammatory cytokines such as IL-6 and IL-1β, among others [21,34,35]. Furthermore, in a study by Kim et al. [36], dietary *Allium* extracts, including PTSO, were found to positively modulate gene expression profiles related to the inflammatory response in alveolar macrophages of pigs experimentally infected with porcine reproductive and respiratory syndrome virus (PRRSV). Similarly, the immunomodulatory capacity of onion thiosulfinates and thiosulfonates has been previously described, contributing to the reduction of certain pro-inflammatory cytokines including IL-1B, IL-6 in preclinical murine models of obesity and inflammatory bowel disease [34]. Additionally, predominant compounds in onion such as quercetin have been described for their anti-inflammatory capacity and COVID-19 protection [37]. Ultimately, it has been shown that *Allium* species could be used for the prevention of SARS-CoV-2 infection, due to its modulatory potential on inflammatory cytokine secretion, phagocytosis, macrophage activation, and immunoglobulin production [38,39,40,41].

Another explanation that could account for the action of these compounds might be related to their ability to modulate the gut microbiota. It is known that the human gut microbiome is integral to immune system modulation and has significant implications for the prognosis of infectious diseases, including respiratory illnesses. In COVID-19, alterations in gut microbiota composition have been linked to disease severity, where dysbiosis can exacerbate systemic inflammation and worsen clinical outcomes. Patients with severe COVID-19 often show a depletion of beneficial gut bacteria with immunomodulatory potential [42]. Modulating the gut microbiota through dietary interventions could enhance the immune response and reduce the severity of infections, particularly in respiratory diseases, where the gut–lung axis is crucial in immune system interactions [43]. Previous studies have demonstrated that the propiin derivatives PTS and PTSO can modulate gut microbiota by enhancing microbial diversity, increasing beneficial bacteria, and reducing harmful pathogens [11]. In preclinical models of (DSS)-induced colitis, PTSO significantly restored gut microbiota composition by normalizing the Firmicutes/Bacteroidetes ratio [43]. Furthermore, in murine models of enteric infection with *Trichuris muris*, PTSO modulated the gut microbiota, leading to reduced inflammatory responses and improved intestinal barrier function [44]. Additionally, the potential of these propiin-derived compounds to modulate the gut microbiota has been extensively studied in farm animals. In poultry, dietary inclusion of these compounds increased beneficial gut bacteria and reduced pathogens, resulting in better growth performance and enhanced resistance to infections [45]. Similarly, in piglets, these compounds improved gut microbiota diversity and increased populations of *Lactobacillus*, which are crucial for maintaining gut health and preventing enteric diseases [46]. These findings highlight the ability of these compounds to modulate the gut microbiota and enhancing immune response.

In our study, patients in the treatment groups were followed up to monitor possible adverse effects from the consumption of the product. No mild or severe adverse effects were observed in any of the volunteers. Similarly, weight and blood pressure were also monitored, and no significant changes were observed. This suggests that the product is safe with a good adverse effect profile. The safety of some compounds present in *Alliaceae* has already been proven both in in vitro studies [47] and in animal models [48], demonstrating the absence of toxicity and mutagenic effects. Also, the safety of extracts from garlic and onion has been studied in patients with COVID-19, with only mild gastrointestinal effects observed in some patients [28].

The effects observed in this study could support the hypothesis of the use of garlic and onion extract products as a complementary approach to the treatment and/or prevention of symptoms caused by SARS-CoV-2 infection, as well as other respiratory infections caused by viruses. The use of such products could have positive implications for public health strategies and economic outcomes by reducing the spread of the virus and allowing faster reintegration into working life.

Our study’s limitations include its retrospective design and the small sample size, which may affect the generalizability of the findings. Furthermore, the homogeneous nature of the study population, in terms of lifestyle habits and residing in a single nursing home, may limit the applicability of the results to broader, more diverse populations. Future research should aim to replicate these findings in larger, multicenter trials and explore the mechanisms underlying the observed effects, particularly the potential immunostimulatory properties of *Allium*-derived compounds.

## 5. Conclusions

In conclusion, this retrospective study built on previous research provides preliminary evidence about the impact of concentrated garlic and onion extract on COVID-19 incidence and severity among elderly nursing home residents, particularly during the seventh pandemic wave marked by the Omicron variant. The findings suggest that these *Allium*-derived compounds may reduce the number of symptoms caused by the SARS-CoV-2 virus and their intensity, although the duration was similar across all groups. These effects are likely due to an immune response-enhancing effect rather than an antiviral effect, given the similar incidence in all groups. The potential public health benefits of these natural compounds warrant more detailed studies, including the analysis of biochemical parameters to explain the mechanism of action of organosulfur compounds from garlic and onion in the context of current and future viral pandemics.

## Figures and Tables

**Table 1:** COVID-19 cases.

Group	Number of Events			Statistic Analysis			
	0	1	2	Ratio ns/s	*p*-Value	IRR c/t	*p*-Value
**Control**	22	9	2	0.012	0.005	3.800	0.032
**Treatment**	29	2	1	0.003	0.002		

Ratio ns/s: ratio no symptoms vs. symptoms. IRR c/t: Incidence rate/ratio control group vs. treatment group.

**Table 2:** Descriptive values of the study volunteers.

	Treatment Group (*n* = 5)	Previously Treated Group (*n* = 6)	Never-Treated GROUP (*n* = 10)	*p* Value
**Age (years)**	84.6 (±11.2)	90.7 (±3.3)	85.3 (±9.5)	0.921
**Weight (Kg)**	63.4 (±7.9)	59.2 (±8.4)	63.3 (±18.1)	0.921
**Gender**				0.202
Male	1 (20%)	0 (0%)	3 (30%)	
Female	4 (80%)	6 (100%)	7 (70%)	
**Tobacco**				0.577
Non smoker	5 (100%)	6 (100%)	10 (100%)	
Smoker	0 (0%)	0 (0%)	0 (0%)	
**Alcohol**				0.577
No drinker	5 (100%)	6 (100%)	10 (100%)	
Drinker	0 (0%)	0 (0%)	0 (0%)	
**Physical activity**				0.136
Very little	2 (40%)	4 (67%)	8 (80%)	
Little	3 (60)	2 (33%)	2 (20%)	

Continuous variables are presented as mean ± standard deviation and categorical variables as *n* (%). The *p* value indicates statistical differences between groups.

**Table 3:** Analysis between groups of incidence and symptoms.

	Group in Treatment	Previously Treated Group	Never Treated Group	*p* Value
**PCR positive/PCR negative**	5/20 ^a^	6/19 ^a^	10/43 ^a^	0.793
**Total symptoms/median**	2/0.0 ^a^	26/4.0 ^b^	44/4.5 ^b^	0.016
**Maximum days of symptoms (mean)**	6.00 ^a^	7.00 ^a^	7.25 ^a^	0.881
**Positive days (mean)**	7.60 ^b^	4.84 ^a^	8.50 ^b^	0.000

*p* value indicates the effect of treatment (Kruskal-Wallis test for total symptoms and ANOVA for times; comparison of treatments using H and Tukey tests). ^a^ and ^b^ indicate statistical similarity or difference between groups.

## Data Availability

The research data for this study have been obtained by Carlos Gracián from the electronic file that Residencia Claret de Granada has. Since it is a confidential file, any questions about the study data should be made to him.
